# Visual Abnormalities Associate With Hippocampus in Mild Cognitive Impairment and Early Alzheimer's Disease

**DOI:** 10.3389/fnagi.2020.597491

**Published:** 2021-01-22

**Authors:** Aonan Zhao, Fang Fang, Binyin Li, Yan Chen, Yinghui Qiu, Yanli Wu, Wei Xu, Yulei Deng

**Affiliations:** ^1^Department of Neurology, Institute of Neurology, Ruijin Hospital, Shanghai Jiao Tong University School of Medicine, Shanghai, China; ^2^Department of Geriatrics, Ruijin Hospital, Shanghai Jiao Tong University School of Medicine, Shanghai, China; ^3^Department of Ophthalmology, Ruijin Hospital, Shanghai Jiao Tong University School of Medicine, Shanghai, China; ^4^Department of Neurology, Ruijin Hospital, Luwan Branch, Shanghai Jiao Tong University School of Medicine, Shanghai, China

**Keywords:** Alzheimer's disease, mild cognitive impairment, hippocampus, visual abnormalities, P100 amplitude, m-RNFL thickness

## Abstract

**Background and Objective:** Alzheimer's disease (AD) has been shown to affect vision in human patients and animal models. This study was conducted to explore ocular abnormalities in the primary visual pathway and their relationship with hippocampal atrophy in patients with AD and mild cognitive impairment (MCI). The aim of this study was to investigate the potential value of ocular examinations as a biomarker during the AD progression.

**Methods:** Patients with MCI (*n* = 23) or AD (*n* = 17) and age-matched cognitively normal controls (NC; *n* = 19) were enrolled. Pattern visual-evoked potentials (PVEP), flash electroretinogram (FERG) recordings and optical coherence tomography (OCT) were performed for all participants. Hippocampal volumes were measured by 3T magnetic resonance imaging. Cognitive function was assessed by Mini Mental State Examination (MMSE), Montreal Cognitive Assessment (MoCA) and Alzheimer's Disease Assessment Scale-cognitive subscale (ADAS-cog). Pearson correlation was employed to analyze the potential associations between ocular abnormalities and hippocampal volumes. Hierarchical regression models were conducted to determine associations between cognitive performances and ocular abnormalities as well as hippocampal volumes after adjusting for confounding factors including age, sex, cognitive reserve, and APOE4 status.

**Results:** PVEP amplitude of P100 waveform was significantly decreased in AD patients compared to MCI and normal individuals. In FERG test, delayed latencies of rod response, rod cone response and 3.0 flicker time were found in cognitively impaired groups, indicating dysfunctions of both the rod and cone systems in the disease progression. OCT test revealed reduced macular retinal nerve fiber layer (m-RNFL) thickness in MCI and AD patients, which significantly correlated with brain structure of hippocampus particularly vulnerable during the progression of AD. Interestingly, P100 amplitude showed a significant association with hippocampal volumes even after adjusting confounding factors including age, sex, and cognitive reserve. Hierarchical regression analysis further demonstrated that m-RNFL thickness, as well as hippocampal volumes, significantly associated with ADAS-cog scores.

**Conclusion:** P100 amplitude and m-RNFL thickness showed significant correlations with brain structure involved in AD-related neurodegeneration, and therefore proved to be potential indicators of brain imaging pathologies.

## Introduction

Alzheimer's disease (AD) is the most common cause of degenerative dementia in older people (Livingston et al., 2020). The early symptoms begin with short-term memory loss and gradually progress to severe impairment in memory, thinking and behavior (Chan et al., 2013). Mild cognitive impairment (MCI) is defined as an early stage of dementia and more than 50% of MCI patients progress to AD in a period of 4–5 years (Petersen, 2006). Therefore, screening, diagnosis and targeted treatment in this stage to prevent conversion to AD is of great importance. Various studies have attempted to identify and evaluate biomarkers for AD and MCI, including state-of-the-art neuroimaging techniques and biochemical analysis of the cerebrospinal fluid. However, time-consuming, radiation, high cost, and invasiveness have hampered their widespread availability.

In recent years, ocular tests have received substantial attention from the scientific community for ocular structural and functional changes in AD through non-invasive and inexpensive evaluation (Heaton et al., 2015). Lots of evidence indicates that visual disturbance is common for AD patients (Cormack et al., 2000). In some AD patients, visual disturbances present as the initial complaint, such as impairment in visual acuity, spatial contrast sensitivity, color sensitivity, and blurred vision (Croningolomb, 1995; Armstrong, 1996). Quantitative data supports the assessment that in the primary visual pathway including retina and lens amyloid-beta accumulation, retinal nerve fiber layer loss, and visual cortex changes can be valuable for the diagnosis of AD (Goldstein et al., 2003; Ohno-Matsui, 2011; Ikram et al., 2012). Optical coherence tomography (OCT) is a non-invasive imaging technique that captures high-resolution and three-dimensional images of the retina. Previous studies in AD patients demonstrated that the retinal nerve fiber layer (RNFL) thickness was attenuated in comparison to healthy controls (Iseri et al., 2006). In addition, the decrease in parapapillary and macular RNFL thickness, and macular volume were related to the cognitive ability of AD patients (Ikram et al., 2012). Other than structural measures, flash electroretinogram (FERG), and pattern visual evoked potential (PVEP) are classic tests to assess full retinal function and retinal ganglion cells, respectively. Abnormal changes are reported in FERG and PVEP in early AD patients as well (Krasodomska et al., 2010).

In this study, we aim to perform ophthalmological examinations including PVEP, FERG and OCT tests to explore ocular functional and structural changes in patients with AD or MCI and age-matched cognitively normal individuals. Since the hippocampus is well-known to be involved in neurodegenerative processes and atrophy of hippocampus predicts the conversion from MCI to AD (Erten-Lyons et al., 2006), we investigated hippocampal volumes by MRI and attempt to identify the relationship between hippocampal volumes and visual system impairment. Our research's goal was to evaluate the potential availability of the application of non-invasive, cost-effective ophthalmic screening tests for AD.

## Materials and Methods

### Patients Enrolment

Participants (*N* = 59) in this study were enrolled at the neurology clinic of Ruijin Hospital affiliated to the Shanghai Jiao Tong University School of Medicine, Shanghai, China, from September 2016 to December 2020. All volunteers gave their informed, written consent prior to study participation. This study was approved by the Research Ethics Committee of Ruijin Hospital. All patients with AD dementia were diagnosed as probable AD dementia following the National Institute on Aging and Alzheimer's Association (NIA-AA) diagnostic guidelines for probable AD dementia with support of structural MRI images (McKhann et al., 2011). To ensure volunteers understood the task, only patients with mild to moderate AD dementia [24 ≥ Mini Mental State Examination (MMSE) ≥ 10] participated on the tests. MCI with deficits in memory function were diagnosed according to the Mayo Clinic criteria (Petersen, 2004). The criteria include subjective memory complaint corroborated by an informant together with preserved everyday activities, a memory impairment based on a standard neuropsychological test, preserved global cognitive functions and finally the exclusion of dementia. Age- and education-adjusted scores falling 1.5 standard deviations below that expected for age and education level may indicate MCI but these are considered as guidelines rather than diagnostic cut-offs. The cognitively normal participants were age-, sex-, and education-matched and were recruited from the local community in Shanghai. Inclusion criteria for normal controls required a MMSE score ≥ 28 without any memory-related complaint. Participants with the presence of dementia or other neurological diseases such as Parkinson's disease were excluded. Besides, individuals were excluded if they have any of the following medical problems: acute diabetic complications, history of acute cerebrovascular accident, history of acute cardiovascular accident, systemic disorders such as malignancy and lupus which were not cured, severe infection, drug abuse or dependency condition and severe psychiatric disorders which were not cured. Individuals with any ocular disease, high refractive error, systemic disease affecting vision or history of ophthalmic surgery were excluded.

### Neuropsychological Assessment

Each participant received a detailed neuropsychological assessment by a memory-related specialist. Additionally, patients with vascular dementia, mental disorders, other neurological diseases, and history of alcohol or drug abuse were also excluded. Specialists assessed cognitive ability in all participants utilizing Mini-Mental State Examination (MMSE, developed by Zhang et al., 1990), Montreal Cognitive Assessment (MoCA) Beijing version, Self-rating depression scale (SDS), Self-rating anxiety scale (SAS), and the 12-item Chinese version of Alzheimer's Disease Assessment Scale-cognitive subscale (ADAS-cog). The cut point established for the MMSE that defines “Alzheimer's disease” was set at 24 (Lopez et al., 2005). We used MoCA cut-off scores of ≤24 points for MCI and ≤22 points for dementia and for individual within 6 years or fewer of formal education, one point was added to the score as a correction (Goldstein et al., 2014). The clinical cut-off raw scores for SAS and SDS were set at 36 and 40, respectively (Dunstan and Scott, 2018). The optimal cut-off score for ADAS-Cog 12-item scale to discriminate between MCI and mild AD was ≥21 (Zainal et al., 2016).

### Optical Coherence Tomography

All participants underwent the OCT examination using the STRATUS OCT Model 3000 (Carl Zeiss Meditec, Inc., Dublin, California, USA). The retinal mapping software was used to calculate the average retinal thickness of the central ring. All eyes were scanned in a radial-spoked pattern centered on the foveola with the scan length of 6 mm. The mean and standard deviations of the macular RNFL (m-RNFL) were calculated in superior, inferior hemiretina. These measurements were performed out to one disc diameter inferiorly and superiorly to the fovea. Optic disc head scans were recorded with optimized z-offset and polarization. The Fast Optic Disc Scan Protocol (OCT-DISC) was used. The software determined automatically the disc margin setting a point at the edge of the retinal pigment epithelium (RPE)/choriocapillaris layer on each side of the disc along a cross-section. The operator was permanently monitoring for steady eye fixation, correct scan position, and good signal-to-noise ratio. Scans were repeated until an image of satisfying quality was obtained.

### Pattern Visual Evoked Potential

All the PVEP examinations (UTAS-E3000, LKC Technologies Inc., Gaithersburg, USA) were performed in an electrically shielded room. Cup-shaped electrodes of Ag/AgCl were placed according to the International 10/20 system (Odom et al., 2004) at the following positions: active electrode in Oz, reference electrode in Fz, ground electrode on earlobes. The recordings were performed in a quiet and dimmed room. The electrical potentials of the occipital cortex were recorded while the participant was looking at the fixation point in the middle of the moving checkboard patterns on the screen one meter in front. The luminance of the white areas was 80 cd m^−2^, and the contrast between the checks was 100%. The checkerboard pattern reversed at the rate of 2 Hz and the viewing distance was 100 cm, the check edges subtended at 15′of visual angle. The signals were fed into an amplifier with the low frequency cut-off filter set at 1.0 Hz and the high frequency cut-off filter set at 100 Hz. The amplified responses were fed to a signal-averaging computer, and 100 responses were averaged with an analysis time of 500 ms. The impedance was kept below 5 kOhm. An alternant checkboard pattern was used as a stimulating pattern and the check size was 23 min. There are three separate phases in the VEP waveform: an initial negative deflection (N75), a prominent positive deflection (P100), and a later negative deflection (N135). The peak latency and peak to peak amplitudes of these waves are measured (Mishra and Kalita, 2004). N75 reflects the activity of fovea and primary visual cortex. P100 originates from dorsal and ventral extrastriate cortex and represents the processing of stimulus characteristics and visuospatial selection (Di Russo et al., 2005; Hamilton et al., 2020). The latency and amplitude of N75 and P100 were calculated in this study.

### Flash Electroretinogram

All participants underwent the FERG examinations using LKC UTAS-E 3000 system (LKC Technologies, Inc., Gaithersburg, USA). The white flash illumination was provided by Ganzfeld 2503D stimulator (LKC Technologies, Inc., Gaithersburg, MD, USA). The software LKC EM for Windows (EMwin v3.0; LKC Technologies, Inc., Gaithersburg, MD, USA) was used to control the recording setting and analyze the data. Signal amplification, luminance calibration, and bandpass filtering were integrated into the LKC system. Standard Ganzfeld scotopic (dark-adapted) and photopic (light- adapted) ERGs were obtained by specialists after pupillary dilation. Subjects took 10 min of light adaptation before recording light-adapted ERGs and were dark adapted for at least 30 min before dark-adapted ERGs were obtained. Topical anesthesia was used for contact lens electrodes. Electroretinographic waveforms were simultaneously obtained from each eye by positioning the active electrodes (ERG-jet monopolar contact lens electrodes; UniversoPlastique SA, Le Cret-Du-Locle, Switzerland) on each cornea. The reference and ground electrodes (Grass subdermal needle electrode; Astro-Med, Inc., West Warwick, RI) were placed subcutaneously in the pinna.

Three ERG recordings were obtained per stimulus intensity. The scotopic and photopic white flash stimuli were performed with an intrastimulus interval of 1 min. The intrastimulus background recovery interval allowed the retina to recover from the previous flash. Photopic tests included: a 100 Td-s (~4 cd-s/m2 [assuming a 6 mm pupil diameter]) flash stimulus presented at a 1 Hz repetition rate with no background luminance (P1); a 58 Td-s (~2 cd-s/m2 [assuming a 6 mm pupil diameter]) red stimulus presented at 3.4 Hz, with a 380 Td blue background (P2); a 100 Td-s (~4 cd-s/m2 [assuming a 6 mm pupil diameter]) flash stimulus presented at a 2 Hz repetition rate with a 340 Td background (P3); and an 85 Td-s (~3 cd-s/m2 [assuming a 6 mm pupil diameter]) flickering (at 28.3 Hz) stimulus (PF). For scotopic tests, stimulus intensity increased by a factor of 10 for each trial, beginning with a 2.8 Td-s (~0.10 cd-s/m2 [assuming a 6 mm pupil diameter]) flash stimulus presented at 0.25 Hz (S1), then a 28 Td-s (~1 cd-s/m2 [assuming a 6 mm pupil diameter]) flash stimulus presented at 0.1 Hz (S2), and finally, a 280 Td-s (~10 cd-s/m2 [assuming a 6 mm pupil diameter]) flash stimulus presented at 0.05 Hz (S3). Flicker ERG was obtained under the same conditions of light adaptation as the light-adapted ERG. And flashes were presented at a rate of ~30 stimuli per second.

### Magnetic Resonance Imaging

MRI Scans were performed on a 3.0T EXCITE HD MR imaging system (Echo-speed plus, General Electric, Milwaukee, WI, USA) with an 8NVHEAD-A coil using a three- dimensional, spoiled gradient recalled echo 3D-SPGR-T1 weighted sequence using the following parameters: repetition time (TR) = 7.7 ms; echo time = 1.6 ms; flip angle = 15′; number of excitations = 1; section thickness = 0.5 mm; field of view = 240 × 240 mm; and matrix size = 256 × 256 mm. The voxel size was 1 × 1 × 1 mm. The voxel-based morphometry was used to analyze the hippocampal volume, with the Biological Parametric Mapping (WFU PickAtlas Tool, http://fmri.wfubmc.edu/) and the MATLAB platform (version 7.0, Mathworks Inc. Sherbom, MA, USA) was used to calculate the hippocampal structure volume.

### Statistical Analysis

Both eyes for each subject were examined and the average of these measurements was taken for the both eyes. Hippocampal volumes of both sides were obtained and the mean value of both sides was included in the analysis. Statistical analysis was performed using SPSS Version 26.0 (SPSS Inc., Chicago, IL, USA) and *P* < 0.05 was considered significant. One-way ANOVAs were used to compare group differences in demographic and clinical variables between the three groups (AD, MCI, and cognitively normal control). We used chi-squared and split chi-squared tests to identify differences in sex and APOE 4 carrier status between the three groups. The Pearson correlation was used to determine the associations between hippocampal volume and ocular indexes, while the partial correlation coefficient (*r*) and associated probability (*P*) were calculated using partial correlation analysis after adjusting for confounding factors, such as age, sex, and years of education. We performed a Bonferroni correction by dividing the critical *P*-value by the number of comparisons being made. For multiple comparisons of demographic characteristics, ocular measurements and hippocampus volumes among AD, MCI, and cognitively normal group, the adjusted *P*-value required for significance is 0.05/3 = 0.017. For correlation analysis, *P*-value is adjusted at 0.05/5 = 0.01. We conducted hierarchical multiple stepwise linear regression analysis to evaluate the possible factors affecting the scores of ADAS-cog, MMSE and MoCA, including age, sex, years of education, APOE 4 carrier status, ophthalmologic measurements and hippocampal volumes. For each model, the regression coefficients (β), R-squared (*R*^2^), change of R^2^ (Δ*R*^2^), and change of *F* (Δ*F*) were calculated, respectively.

## Results

In this research, 19 cognitively normal controls, 23 MCI patients and 17 AD patients were included. There was no significant difference in age, sex, years of education and *APOE* 4 carrier status among three groups. MMSE, MoCA and ADAS-cog were performed to assess cognitive function for all participants, and the scores of which revealed significantly typical trends among groups as expected (*P* < 0.001). Detailed demographic characteristics of each group are shown in Table 1.

**Table 1:** Demographic characteristics of all participants.

**Demographics**	**NC**	**MCI**	**AD**	***P*-value**
N	19	23	17	–
Age, y, (SD)	66.63 (6.17)	68.43 (5.70)	70.24 (7.53)	0.251
Sex M/F	8/11	12/11	9/8	0.756
Education, y, (SD)	10.74 (3.00)	12.78 (3.48)	10.47 (3.47)	0.057
ApoE4 carrier (%)	15.8%	30.4%	41.2%	0.238
MMSE (SD)	28.79 (1.03)	26.91 (1.47)^#^	21.18 (3.09)^*^^§^	<0.001
MoCA (SD)	24.89 (2.13)	20.57 (2.21)^#^	15.65 (2.81)^*^^§^	<0.001
ADAS-cog (SD)	14.21 (4.57)	18.00 (3.22)^#^	31.88 (4.31)^*^^§^	<0.001
SAS (SD)	28.26 (5.11)	27.26 (4.87)	27.64 (4.78)	0.789
SDS (SD)	27.47 (6.44)	29.96 (6.15)	29.76 (6.10)	0.389

*AD, Alzheimer's disease; MCI, mild cognitive impairment; NC, normal control; M, male; F, female; y, years; SD, standard deviation; MMSE, Mini-Mental State Examination; MoCA, Montreal Cognitive Assessment; ADAS-cog, Alzheimer's Disease Assessment Scale-cognitive subscale; SAS, Self-rating anxiety scale; SDS, Self-rating depression scale*.

# *MCI vs. NC, P < 0.017*.

* *AD vs. MCI, P < 0.017*.

§ *AD vs. NC, P < 0.017*.

Results of ocular measurements are listed in Table 2. Of the PVEP waveform components recognized, AD patients showed a decrease in the P100 amplitudes when compared to MCI group (*P* = 0.016), while comparison of the N75 wave revealed no statistically significant differences for the amplitudes or the latencies. In the FERG tests, eyes of AD patients had significantly prolonged rod response latency time when observed in the dark-adapted environment in comparison with age-matched cognitively normal controls (*P* = 0.013). As light stimulus in darkness was intensified to activate the cone system and mixed responses from both rod and cone systems were recorded, delayed rod cone response latency time was found in both MCI (*P* = 0.003) and AD (*P* = 0.003) patients compared to normal controls. In the light environment, where responses from the cone system were recorded, AD patients showed longer reaction time reflected as increased 3.0 flicker latencies when compared to MCI patients (*P* = 0.013) and healthy participants (*P* = 0.008). However, no statistically significant group difference was found in amplitudes of all responses. In addition, structural changes were examined by OCT tests. Remarkably reduced thickness of m-RNFL was observed in AD eyes than that in MCI eyes (*P* = 0.014) and NC eyes (*P* < 0.001). MCI patients showed significantly thinner m-RNFL compared to normal individuals (*P* = 0.011) as well. However, no significant morphological changes in the optic disc head were found for the rim area, disc area, and cup volume.

**Table 2:** PVEP, FERG, OCT results, and Hippocampal volumes for all participants.

**Variable**	**NC**	**MCI**	**AD**	***P*-value**	***F*-value**	**Total *df***	**Effect size ($\eta_{p}^{2}$)**
N75 latency(ms), (SD)	63.84 (13.49)	63.76 (17.70)	62.15 (16.30)	0.938	0.064	58	0.003
N75 amplitude(μV), (SD)	2.41 (0.62)	2.32 (0.85)	2.48 (0.87)	0.808	0.214	58	0.027
P100 latency(ms), (SD)	99.89 (8.31)	104.33 (10.69)	104.65 (14.54)	0.354	1.058	58	0.054
P100 amplitude(μV), (SD)	6.23 (2.71)	6.92 (3.48)	4.61 (2.17)^*^	0.050	3.159	58	0.099
Rod response latency(ms), (SD)	49.93 (6.63)	51.55 (11.45)	59.47 (9.76)^§^	0.010	5.028	58	0.149
Rod response amplitude(μV), (SD)	90.69 (9.47)	88.95 (11.17)	92.68 (10.33)	0.537	0.629	58	0.025
Rod cone response latency(ms), (SD)	41.36 (7.21)	50.50 (10.52)^#^	50.82 (10.65)^§^	0.002	6.859	58	0.237
Rod cone response amplitude(μV), (SD)	110.63 (36.95)	130.14 (42.17)	108.97 (26.95)	0.125	2.157	58	0.048
Cone response latency(ms), (SD)	16.85 (2.12)	15.70 (2.58)	16.43 (2.97)	0.345	1.084	58	0.029
Cone response amplitude(μV), (SD)	93.47 (16.24)	90.50 (20.53)	91.84 (16.91)	0.871	0.138	58	0.008
3.0 flicker latency(ms), (SD)	25.85 (4.11)	26.31 (5.90)	30.63 (5.72)^*^^§^	0.015	4.554	58	0.121
3.0 flicker amplitude(μV), (SD)	75.09 (13.58)	68.96 (20.96)	73.73 (15.00)	0.482	0.740	58	0.018
m-RNFL thickness(μm), (SD)	96.76 (7.30)	88.65 (9.81)^#^	80.46 (8.37)^*^^§^	<0.001	15.94	58	0.393
Rim area(mm^2^), (SD)	1.36 (0.14)	1.44 (0.32)	1.50 (0.24)	0.255	1.40	58	0.044
Disc area(mm^2^), (SD)	2.08 (0.25)	1.98 (0.33)	2.09 (0.32)	0.441	0.831	58	0.055
Cup volume(mm^3^), (SD)	0.30 (0.04)	0.28 (0.08)	0.30 (0.07)	0.476	0.752	58	0.020
Hippo volume(cm^3^), (SD)	2.53 (0.45)	1.84 (0.30)^#^	1.33 (0.37)^*^^§^	<0.001	46.91	58	0.636

*PVEP, pattern visual evoked potential; FERG, flash electroretinogram; OCT, optical coherence tomography; AD, Alzheimer's disease; MCI, mild cognitive impairment; NC, normal control; SD, standard deviation; m-RNFL, macular retinal nerve fiber layer; Hippo, hippocampus*.

# *MCI vs. NC, P < 0.017*.

* *AD vs. MCI, P < 0.017*.

§ *AD vs. NC, P < 0.017*.

**Figure 1:**
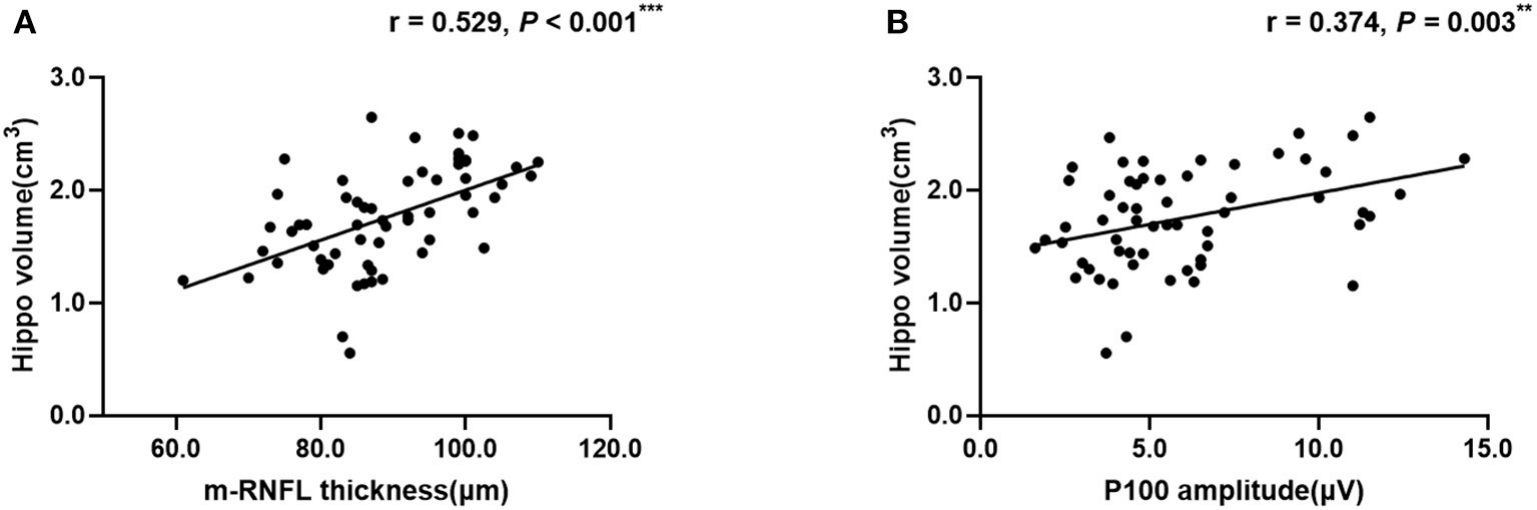
Correlation analysis between hippocampal volume and m-RNFL thickness **(A)** or P100 amplitude **(B)**. The parameter was estimated using Pearson's correlation coefficients. Hippo, hippocampus; m-RNFL, macular retinal nerve fiber layer. ***P < 0.001, **P < 0.01.

Regarding hippocampal volumes investigated by MRI, as shown in Table 2, significantly decreased size of hippocampus was found in patients with AD when compared to MCI group (*P* < 0.001) and healthy individuals (*P* < 0.001). A comparison of the MCI group with the NC group showed obviously shrunken structure of hippocampus as well (*P* < 0.001). Then we performed Pearson correlation analysis to explore associations between hippocampal volumes and ocular abnormalities as described in PVEP, FERG, and OCT examinations, respectively. As shown in Figure 1, m-RNFL thickness (*r* = 0.529, *P* < 0.001, Figure 1A) and P100 amplitude (*r* = 0.374, *P* = 0.003, Figure 1B) significantly correlated with hippocampal volumes. With adjustments made for the confounding factors of age, sex, years of education and *APOE* 4 carrier status, as shown in Table 3, associations remained significant between the volumes of hippocampus and m-RNFL thickness (*r* = 0.521, *P* < 0.001) as well as P100 amplitude (*r* = 0.389, *P* = 0.003). We did not find any significant associations between rod response latency, rod cone response latency, 3.0 flicker latency and the brain structure of hippocampus.

**Table 3:** Partial correlation analysis between visual indexes and hippocampal volume.

**Variable**	***r-*value**	***P*-value**
m-RNFL thickness	0.521	<0.001^*^
P100 amplitude	0.389	0.003^*^
Rod response latency	−0.183	0.178
Rod cone response latency	0.078	0.570
3.0 flicker latency	−0.065	0.634

*m-RNFL, macular retinal nerve fiber layer*.

*The partial correlation coefficient of the correlation analysis was corrected with confounding factors including age, gender, and years of education*.

* *P < 0.01*.

A hierarchical regression analysis was further conducted to determine the associations between ocular indexes, hippocampal volumes and cognitive function. After controlling for age, sex, years of education and *APOE* 4 carrier status, hippocampal volumes (β = −0.490, *P* < 0.001) and m-RNFL thickness (β = −0.242, *P* = 0.031) were significantly associated with ADAS-cog scores (Table 4). Regarding scores of MMSE (Table 5) and MoCA (Table 6), however, just the parameter of hippocampal volumes demonstrated strongly positive associations (β = 0.527, *P* < 0.001; β = 0.664, *P* < 0.001; respectively), while parameters of ocular indexes revealed no significant association (*P* > 0.05).

**Table 4:** Hierarchical regression analysis for ADAS-cog score in all participants.

**Variable**	**Regression 1**	**Regression 2**	**Regression 3**
Age	0.141	0.032	−0.061
Gender	−0.068	−0.147	−0.111
Years of education	−0.205	−0.219	−0.167
ApoE4 status	0.146	0.090	0.083
Hippo volume		−0.667^***^	−0.490^***^
m-RNFL thickness			−0.242^*^
P100 amplitude			−0.028
Rod response latency			0.204
Rod cone response latency			0.044
3.0 flicker latency			0.173
*R^2^*	0.107	0.528	0.635
Δ*R^2^*	0.107	0.421	0.106
Δ*F*	1.625	47.262^***^	2.794^*^

*ADAS-cog, Alzheimer's Disease Assessment Scale-cognitive subscale; Hippo, hippocampus; m-RNFL, macular retinal nerve fiber layer*.

*** P < 0.001,

* P < 0.05.

**Table 5:** Hierarchical regression analysis for MMSE score in all participants.

**Variable**	**Regression 1**	**Regression 2**	**Regression 3**
Age	−0.006	0.095	0.219
Gender	−0.113	−0.040	−0.054
Years of education	0.218	0.230	0.159
ApoE4 status	−0.188	−0.136	−0.139
Hippo volume		0.617^***^	0.527^***^
m-RNFL thickness			0.134
P100 amplitude			−0.004
Rod response latency			−0.177
Rod cone response latency			−0.128
3.0 flicker latency			−0.022
*R^2^*	0.076	0.437	0.491
Δ**R*^2^*	0.076	0.361	0.054
Δ*F*	1.115	33.942^***^	1.024

*MMSE, Mini-Mental State Examination; Hippo, hippocampus; m-RNFL, macular retinal nerve fiber layer*.

*** *P < 0.001*.

**Table 6:** Hierarchical regression analysis for MoCA score in all participants.

**Variable**	**Regression 1**	**Regression 2**	**Regression 3**
Age	−0.041	0.068	0.068
Gender	−0.078	0.001	−0.075
Years of education	0.007	0.021	0.046
ApoE4 status	−0.191	−0.135	−0.108
Hippo volume		0.664^***^	0.664^***^
m-RNFL thickness			0.085
P100 amplitude			−0.170
Rod response latency			−0.050
Rod cone response latency			0.025
3.0 flicker latency			−0.123
*R^2^*	0.042	0.460	0.506
Δ**R*^2^*	0.042	0.417	0.047
Δ*F*	0.597	40.957^***^	0.905

*MoCA, Montreal Cognitive Assessment; Hippo, hippocampus; m-RNFL, macular retinal nerve fiber layer*.

*** P < 0.001.

## Discussion

This study performed ophthalmic measurements including PVEP, FERG and OCT tests to investigate functional and structural changes of retina and/or visual pathway in MCI and AD patients compared to cognitively normal aging. Our results demonstrated that PVEP amplitude of P100 waveform was significantly decreased in AD patients compared to MCI and normal individuals. In FERG test, delayed latencies of rod response, rod cone response and 3.0 flicker time were found in cognitively impaired groups, indicating dysfunctions of both the rod, and cone systems in the disease progression. OCT test revealed reduced m-RNFL thickness in MCI and AD patients, which significantly correlated with brain structure of hippocampus particularly vulnerable during the progression of AD. Strikingly, P100 amplitude showed significant association with hippocampal volumes even after adjusting confounding factors including age, sex, and years of education. Hierarchical regression analysis further demonstrated that m-RNFL thickness, as well as hippocampal volumes, had significant predictive value in assessing cognitive performance in terms of neuropsychological test of ADAS-cog.

PVEPs are used to evaluate the functional integrity of the visual pathway. P100, typically peaking at about 100 ms, is the major component of the PVEPs. It is considered to generate from dorsal and ventral extrastriate cortex and represent the processing of stimulus characteristics and visuospatial selection (Di Russo et al., 2005). Extrastriate cortex belongs to part of visual association areas and shows significant microscopic pathology with beta-amyloid (Aβ) aggregates and neurofibrillary tangles in the post-mortem investigation of AD patients (Arnold et al., 1991). High synaptic complexity of the association cortices may enable and amplify the propagation of disease pathology (Mckee et al., 2006). In line with previous reports (Krasodomska et al., 2010; Stothart et al., 2015), we propose that reduced P100 amplitude in AD patients may reflect this pathology in visual association areas and prove a sensitive marker in examining cortical pathology in AD progression beyond currently available behavioral, imaging and biochemical tools. Interestingly, partial correlation analysis revealed that decreased P100 amplitude significantly positively correlated with atrophied volume of hippocampus. Recent findings suggest that hippocampus participates in visuospatial memory formation. Dysfunction of synaptic plasticity in the visual cortex may influence visuospatial information process and thus in turn hippocampal formation (Tsanov and Manahan-Vaughan, 2008). Close relationship between P100 amplitude and hippocampal volumes may strengthen the potential value of PVEP waveform of P100 in early screening and monitoring the disease progression.

FERG provides an objective measure of cellular function in retina. The cellular responses recorded can be specific to rod photoreceptors under dark-adapted conditions or cone photoreceptors in light-adapted environment. In our study, prolonged latencies of rod response and 3.0 flicker time were found in AD patients when compared to MCI group and/or normal aging, while delayed rod cone response latency was observed in both MCI and AD patients compared to healthy controls, indicative of being a potential marker in early stage of cognitive decline. In an animal study of mice carrying *APOE4*, the most prevalent genetic risk factor for AD, ERG recordings revealed significant attenuation of mixed rod cone responses in dark-adapted eyes, which is considered to be related to observed decrease in synaptic density of the retinal synaptic layers (Ran et al., 2013). However, our following correlation analysis demonstrated no significant association between FERG components and hippocampal volumes. In view of recent reports of FERG alterations in other neurodegenerative diseases (Devos et al., 2005; Pearl et al., 2017), more comprehensive longitudinal studies are needed to improve its specificity in clinical practice.

Retina has been viewed as an extension of central nervous system due to their developmental and structural similarities. Histopathological studies reported typical Aβ deposits, loss of retinal ganglion cells and optic nerve degeneration in AD patients, hence initiating in the retrograde damaged ganglion cell fibers and leading to the morphological changes, such as RNFL thinning (Blanks et al., 1996a,b). Consistent with recent evidence (den Haan et al., 2017b; Chan et al., 2018), we have found significantly reduced overall thickness of m-RNFL in AD and MCI patients compared to age-matched control counterparts in the current research. In addition, our results demonstrated a significant correlation between total m-RNFL thickness and hippocampal volumes, corroborating the link between retina and brain in the progression of cognitive impairment. Several studies have investigated the connections between retinal changes and MRI volumetric measurements of brain structures. In early-onset AD patients, den Haan et al. (2017a) found total macular thickness correlated with parietal cortical atrophy, suggestive of reflection of cerebral cortical changes in the retina, independent of AD pathological markers, such as amyloid. In non-demented older adults, thinner overall RNFL and peripapillary RNFL showed good associations with smaller volumes of medial temporal lobes and hippocampus, while no significant associations were found in the control regions, which were located outside the visual pathway and the regions involved in AD (Méndez-Gómez et al., 2018; Shi et al., 2020). In a recent study involving AD and MCI patients, better correlation was observed between inner perifovea retinal thickness and the hippocampal and entorhinal cortical volumes, which are specifically affected in the early stage of AD, although no correlation was found between m-RNFL thickness and cognitive performance, and no significant difference of m-RNFL thinning was shown in patients with MCI compared to those with AD (Tao et al., 2019). In our study, m-RNFL thickness exhibited a significant reduction in MCI patients in comparison to AD patients and presented as an independent predictor of ADAS-cog score, along with average hippocampal volumes, in the hierarchical regression model. The discrepancies may result from high degree of variability in the study inclusion/exclusion criteria, assessment of neuropsychological performance and the rigor of adjustment of confounding factors. However, our results have added more evidence for the availability of application of m-RNFL thickness as a promising biomarker for future AD diagnosis, monitoring, and prognosis.

In the current cross-sectional study we have enrolled relatively small sample size, partially due to strict exclusion criteria of patient enrollment and adaptability to full screen of ocular structural and functional measurements. A longitudinal study with expanded sample size is required to validate the retina-brain association in the neurodegenerative process of cognitive decline. Owing to the lack of Aβ or Tau-related markers, we were unable to analyze the relationship between those pathological markers and ocular measurements, and hence the present findings cannot directly confirm whether the alterations in the ophthalmological examinations are specific to AD. Additionally, quandrant-specific changes of peripapillary RNFL thickness have been reported in recent studies, although results remained controversial (Alber et al., 2020). Evidence from numerous neuropsychological and functional neuroimaging studies suggested that right posterior hippocampus is implicated in visual information and visuospatial memory formation. Changes in the volume of the anterior and posterior hippocampus can be compensated and therefore underestimate the alteration of the hippocampus most involved in vision (Hüfner et al., 2011). Braak's staging of AD showed that neurodegeneration began in entorhinal and perirhinal cortex suggesting the area most and earliest affected by neurofibrillary tangles in Alzheimer's disease, even before the hippocampus (Braak and Braak, 1995). Further detailed studies with investigations of changes of different segmentation of RNFL thickness and more brain regions including entorhinal cortex, perirhinal cortex and subareas of hippocampus are needed to assist in clarification of possible mechanisms underlying retina-brain association in the disease progression of cognitive decline and AD.

In conclusion, our results presented significant alterations in ophthalmological examinations including PVEP, FERG responses and m-RNFL thickness in patients with MCI and AD. P100 amplitude and m-RNFL thickness showed significant correlations with brain structure involved in AD-related neurodegeneration, and therefore proved to be potential indicators of brain imaging pathologies. Future prospective researches are required to determine the reliability of ocular investigations, especially measurement of RNFL thickness during AD progression and recognize whether retina has broad implications in AD pathology to serve as a promising non-invasive and cost-effective biomarker.

## Data Availability Statement

The raw data supporting the conclusions of this article will be made available by the authors, without undue reservation.

## Ethics Statement

The studies involving human participants were reviewed and approved by Ruijin Hospital Ethics Committee, Shanghai Jiao Tong University School of Medicine. The patients/participants provided their written informed consent to participate in this study.

## Author Contributions

YD and WX designed the study, edited the manuscript, and validated the statistics. AZ and FF collected the data, wrote and edited the manuscript, and performed the statistics. YC and YW collected the data and help revised the manuscript. BL and YQ collected the data. All authors contributed to the article and approved the submitted version.

## Conflict of Interest

The authors declare that the research was conducted in the absence of any commercial or financial relationships that could be construed as a potential conflict of interest.

## Abbreviations

AD: Alzheimer's disease
MCI: Mild cognitive impairment
PVEP: Pattern visual-evoked potentials
FERG: Flash electroretinogram
OCT: Optical coherence tomography
MMSE: Mini Mental State Examination
MoCA: Montreal Cognitive Assessment
ADAS-cog: Alzheimer's Disease Assessment Scale-cognitive subscale
m-RNFL: Macular retinal nerve fiber layer
SDS: Self-rating depression scale
SAS: Self-rating anxiety scale
MRI: Magnetic resonance imaging.
